# Primary phagocytosis of viable neurons by microglia activated with LPS or Aβ is dependent on calreticulin/LRP phagocytic signalling

**DOI:** 10.1186/1742-2094-9-196

**Published:** 2012-08-13

**Authors:** Michael Fricker, María José Oliva-Martín, Guy C Brown

**Affiliations:** 1 Department of Biochemistry, University of Cambridge, Tennis Court Road, Cambridge, CB2 1QW, UK; 2 Present address: HMRI, University of Newcastle, Newcastle upon Tyne, NSW, Australia

**Keywords:** Phagocytosis, Neuron, Microglia, Calreticulin, LRP, Inflammation, Amyloid, Neurodegeneration, Cell death, Phagoptosis

## Abstract

**Background:**

Microglia are resident brain macrophages that can phagocytose dead, dying or viable neurons, which may be beneficial or detrimental in inflammatory, ischaemic and neurodegenerative brain pathologies. Cell death caused by phagocytosis of an otherwise viable cell is called ‘primary phagocytosis’ or ‘phagoptosis’. Calreticulin (CRT) exposure on the surface of cancer cells can promote their phagocytosis via LRP (low-density lipoprotein receptor-related protein) on macrophages, but it is not known whether this occurs with neurons and microglia.

**Methods:**

We used primary cultures of cerebellar neurons, astrocytes and microglia to investigate the potential role of CRT/LRP phagocytic signalling in the phagocytosis of viable neurons by microglia stimulated with lipopolysaccharide (LPS) or nanomolar concentrations of amyloid-β peptide_1-42_ (Aβ). Exposure of CRT on the neuronal surface was investigated using surface biotinylation and western blotting. A phagocytosis assay was also developed using BV2 and PC12 cell lines to investigate CRT/LRP signalling in microglial phagocytosis of apoptotic cells.

**Results:**

We found that BV2 microglia readily phagocytosed apoptotic PC12 cells, but this was inhibited by a CRT-blocking antibody or LRP-blocking protein (receptor-associated protein: RAP). Activation of primary rat microglia with LPS or Aβ resulted in loss of co-cultured cerebellar granule neurons, and this was blocked by RAP or antibodies against CRT or against LRP, preventing all neuronal loss and death. CRT was present on the surface of viable neurons, and this exposure did not change in inflammatory conditions. CRT antibodies prevented microglia-induced neuronal loss when added to neurons, while LRP antibodies prevented neuronal loss when added to the microglia. Pre-binding of CRT to neurons promoted neuronal loss if activated microglia were added, but pre-binding of CRT to microglia or both cell types prevented microglia-induced neuronal loss.

**Conclusions:**

CRT exposure on the surface of viable or apoptotic neurons appears to be required for their phagocytosis via LRP receptors on activated microglia, but free CRT can block microglial phagocytosis of neurons by acting on microglia. Phagocytosis of CRT-exposing neurons by microglia can be a direct cause of neuronal death during inflammation, and might therefore contribute to neurodegeneration and be prevented by blocking the CRT/LRP pathway.

## Background

Most neurological diseases involving neuronal loss are accompanied by the appearance of activated microglia in the affected tissues [1]. Microglia are resident brain macrophages that mediate the immune response against CNS infections and clear cellular debris following injury. However, there is growing evidence that inflammatory-activated microglia actively participate in the death of neurons during neurodegenerative processes, for example through release of reactive oxygen and nitrogen species (ROS/RNS) and release of pro-inflammatory neurotoxic cytokines and inflammatory mediators such as TNF-α [2]. We have recently described a novel form of neuronal death mediated by inflammatory-activated microglia in which microglia phagocytose viable neurons, referred to as ‘primary phagocytosis’ or ‘phagoptosis’ [3,4]. Phagocytosis is normally thought to occur after the target cell has undergone cell death, but we found that in inflammatory conditions inhibition of phagocytic signalling rescues neurons both *in vitro* and *in vivo*, demonstrating that phagocytosis can be a direct cause of neuronal death in models of inflammatory neurodegeneration [5-7].

Phagocytosis is controlled by a complex array of signals. The interaction between a number of ‘eat-me’ and ‘don’t-eat-me’ signals located on the target cell surface and their respective receptors on the phagocyte determine whether or not phagocytosis takes place [8]. The best-characterised ‘eat-me’ signal is exposure of the phospholipid phosphatidylserine (PS) on the outer leaflet of the plasma membrane. In most viable cells that are not activated, PS is almost exclusively localised on the inner leaflet of the plasma membrane due to an aminophospholipid translocase that pumps PS from the outer to the inner leaflet. Upon induction of cell death by apoptosis or necrosis, PS becomes exposed on the cell surface due to inactivation of the translocase or activation of a scramblase, which randomises phospholipid distribution between the inner and outer leaflets thus resulting in net PS exposure. However, PS exposure also occurs on the surface of viable cells when ‘activated’, usually as a result of calcium stimulation of the scramblase and inhibition of the translocase, for example during activation of all leucocytes [9-11], and on neurons exposed to oxidants from activated microglia [5]. Exposed PS can be either bound directly by some phagocyte receptors, such as Tim4, stabilin-1 and −2 and BAI1, or bound by bridging proteins such as MFG-E8, which activates phagocytosis via the vitronectin receptor (α_v_β_3/5_ integrin) [8]. Indeed we have shown that primary phagocytosis of viable neurons by inflammatory-activated microglia is mediated by microglia-induced PS exposure on viable neurons, evoking microglial phagocytosis via MFG-E8 and the vitronectin receptor [5,7].

Surface-exposed calreticulin (CRT) has been demonstrated to act as an eat-me signal in a number of cell types [12]. CRT, principally characterized as an endoplasmic reticulum (ER)-resident chaperone, is constitutively expressed at the surface of numerous cancer cell lines and its expression at the cell surface can be increased in the early stages of apoptosis induced by a subset of apoptotic stimuli including anthracyclins and UV irradiation [13,14]. CRT has been shown to act as an essential eat-me signal promoting phagocytosis of apoptotic cells and its activity can be modulated not only by increasing exposure at the cell surface but also potentially by rearrangement of existing exposed CRT [15,16]. Surface-exposed CRT is recognised by the phagocytic receptor LRP (low-density lipoprotein receptor-related protein) [15,17], although CRT is also found associated with LRP on the phagocyte membrane where it acts as a co-receptor for LRP ligands such as C1q and alpha-2-macroglobulin [18]. The constitutive expression of CRT on the surface of a number of cell types does not necessarily result in their phagocytosis as don’t-eat-me signals have a dominant inhibitory effect on phagocytosis, for example CD47 and its receptor SIRPα [13,15,19,20]. The role and regulation of exposed CRT and LRP in mediating phagocytosis of neurons by microglia is unknown. We therefore sought to test the requirement for CRT- and LRP-mediated signalling for phagocytosis of dying neurons and primary phagocytosis of viable neurons in models of inflammatory neurodegeneration. Here we demonstrate that neuronally exposed CRT is required as an eat-me signal for phagocytosis of both apoptotic and viable neurons by microglia, and that CRT is constitutively exposed on the surface of neurons but this only promotes phagocytosis in specific contexts, and indeed released CRT can inhibit phagocytosis at microglia.

## Methods

All experiments were performed in accordance with the UK Animals (Scientific Procedures) Act (1986) and approved by the Cambridge University Local Research Ethics Committee.

### Cell culture and treatments

Mixed neuronal/glial cerebellar cultures were prepared from the cerebella of postnatal day 5 to 7 rats as previously described [21] and were allowed to mature *in vitro* for six to eight days prior to treatment. Pure microglia were prepared from mixed cortical astroglial/microglial cultures as previously described [5]. BV2 microglial cells were grown in Dulbecco’s modified Eagle’s medium (DMEM, Invitrogen, Carlsbad, CA, USA)) supplemented with 10% fetal bovine serum (FBS, PAA Laboratories, Colbe, Germany). PC12 neuronal cells were grown in Roswell Park Memorial Institute medium (RPMI, Invitrogen) supplemented with 10% FBS and 20% horse serum (Sigma-Aldrich, St Louis, MO, USA). PC12 were plated on collagen-coated tissue culture plates (0.5 mg/ml collagen, Sigma-Aldrich). All tissue culture medium was supplemented with 100 units/ml penicillin G, 100 μg/ml streptomycin sulphate (Invitrogen). Reagents were procured as follows: lipopolysaccharide (LPS), calreticulin (CRT), cytochalasin D (CytoD), 5-(and-6)-carboxytetramethylrhodamine succinimidyl ester (TAMRA) were from Sigma-Aldrich. β 1–42 monomers (EZBiolab, Carmel, IN, USA) were prepared as previously described [6]. Receptor-associated protein (RAP, R&D systems, Minneapolis, MN, USA), normal mouse IgG (mIgG, Santa Cruz Biotech, Santa Cruz, CA, USA), anti-CRT antibodies (Abcam, Cambridge, UK; Stressgen, Brussels, Belgium), anti-LRP (American Diagnostica Inc., Stamford, CT, USA), Alexa 488-labelled isolectin B4 (IB4, Molecular Probes, Eugene OR, USA). Neuronal and microglial cell survival was quantified three days after stimulation as previously described [5]. Anti-CRT and anti-LRP blocking antibodies were Fc-blocked with an F(ab’)2 fragment antibody (Jackson Immunoresearch, West Grove, PA, USA). Nitrite levels in culture supernatants were measured as previously described [5].

### BV2 and PC12 phagocytosis assay

BV2 were plated in 6-well plates in DMEM plus 0.5% FBS and were at approximately 60% confluency when target cells were added. PC12 in suspension were stained for 10 minutes with 50 μM TAMRA, washed in warm PBS and then plated in 10 cm collagen-coated dishes at high density. UV-treated PC12 received 200 mJ/cm^2^ irradiation. Untreated and UV-treated PC12 were harvested 16 hours after UV treatment by trypsinisation. PC12 target cells were counted and resuspended in DMEM plus 0.5% FBS. Some 200,000 PC12 target cells were added to each well of BV2 (approximate four-fold excess of target PC12 cells compared to BV2) followed by a two-hour incubation at 37°C. For FACS analysis, BV2 were stained with IB4 (1 μg/ml) for 15 minutes prior to washing in PBS and brief trypsinisation to detach cells. BV2 were then resuspended in 200 μl PBS and FACS analysis performed using an Accuri C6 Flow Cytometer (BD Services, San Jose, CA, USA). Alexa 488 IB4 fluorescence was detected in FL1 channel whilst TAMRA fluorescence was detected in FL2. For fluorescence microscopy, BV2 were labeled with IB4 as above and washed briefly in PBS prior to labelling of nuclear DNA with Hoechst 33342 [5]. Cells were imaged on an Olympus Fluoview 300 microscope (Olympus, Tokyo, Japan).

### Transwell and microglial reconstitution experiments

Following six to seven days *in vitro* microglia were selectively eliminated from cerebellar cultures by adding 50 mM L-leucine methyl ester (LME, Sigma-Aldrich). After three hours LME-containing medium was aspirated, neurons washed once in warm HBSS (Invitrogen) and then medium was replaced with conditioned medium from sister cultures. Twenty-four hours later, 6.5 mm 0.4 μm pore size polycarbonate transwell inserts (Corning, Sigma-Aldrich) that had been poly-L-lysine coated were inserted and 25,000 microglia were plated onto the insert. After 24 hours, LPS was added at 100 ng/ml as indicated in figure legends. Forty-eight hours later, transwell inserts containing microglia were removed. During this time microglia were purified and plated in 6-well plates, left for 24 hours and then incubated for a further 24 hours with 100 ng/ml LPS. LPS-activated pure microglia were gently blown from wells after a brief incubation in Versene solution (Invitrogen) at 37°C. At this point, blocking antibody was added to the neurons or to purified LPS-activated microglia in suspension for one hour at 37°C. Neurons were washed three times in warm HBSS before conditioned medium from untreated sister cultures was added back. LPS-activated microglia were washed three times in warm HBSS, collected by centrifugation and counted. A total of 25,000 LPS-activated microglia was then added directly back to neuronal cultures as indicated and plates were spun briefly to allow microglia to settle. Cerebellar cultures were then incubated for six hours at 37°C before neuronal survival and numbers were assessed as described above. For addition of exogenous CRT, microglia were eliminated from cerebellar granule cells (CGC) that had been *in vitro* for seven days using LME as before. After 24 hours, 1 μg/ml CRT (Sigma-Aldrich) was added directly to neurons and left to incubate for two hours, followed by three washes in warm HBSS. Pure microglia that had either been left untreated or LPS activated for 24 hours as described above were then added back to neurons as indicated at a density of 25,000 cells per well (24-well plate), plates incubated for six hours at 37°C prior to quantification of neuronal number and survival.

### Externalised protein biotinylation and pull-down

Surface biotinylation was performed using the Pierce Cell Surface Protein Isolation Kit including Sulfo-NHS-SS-Biotin as the labelling reagent (Thermo Fisher Scientific, Waltham, MA, USA). Cerebellar cultures were seeded in 6-well plates and after six days in culture microglia were eliminated with LME as described above. After 24 hours, a poly-L-lysine-coated transwell was inserted and 200,000 purified microglia were plated on the transwell. Twenty-four hours later, 100 ng/ml LPS was added where indicated. After 48 hours, further incubation transwells were removed and neuron plates were transferred to ice and washed several times in HBSS prior to addition of Sulfo-NHS-SS-Biotin. All subsequent steps including streptavidin pull-down of biotinylated proteins followed the manufacturer’s instructions. Neurons were lysed in a volume of 500 μl and prior to pull-down a load sample of 40 μl was collected. Biotinylated proteins captured on streptavidin beads were eluted by boiling in SDS gel loading buffer. Proteins were separated by SDS-PAGE and transferred to nitrocellulose membranes for western blot detection as previously described [22].

### Statistical analysis

Statistical analysis was performed using SPSS software. All results represent the mean value from at least three separate experiments (see figure legends) with each individual experiment having two replicates per condition with four fields counted per replicate. In figure legends n = x refers to the number of separate experiments performed. Error bars represent the standard error of the mean of experiments (SEM). Normality of data was verified using the Shapiro-Wilk test. Data was analysed using one-way ANOVA and post hoc Bonferroni test. In figures * = *P* < 0.05, ** = *P* < 0.01, *** = *P* < 0.001.

## Results

### CRT/LRP signalling in phagocytosis of apoptotic PC12 cells by BV2 microglial cells

**Figure 1:**
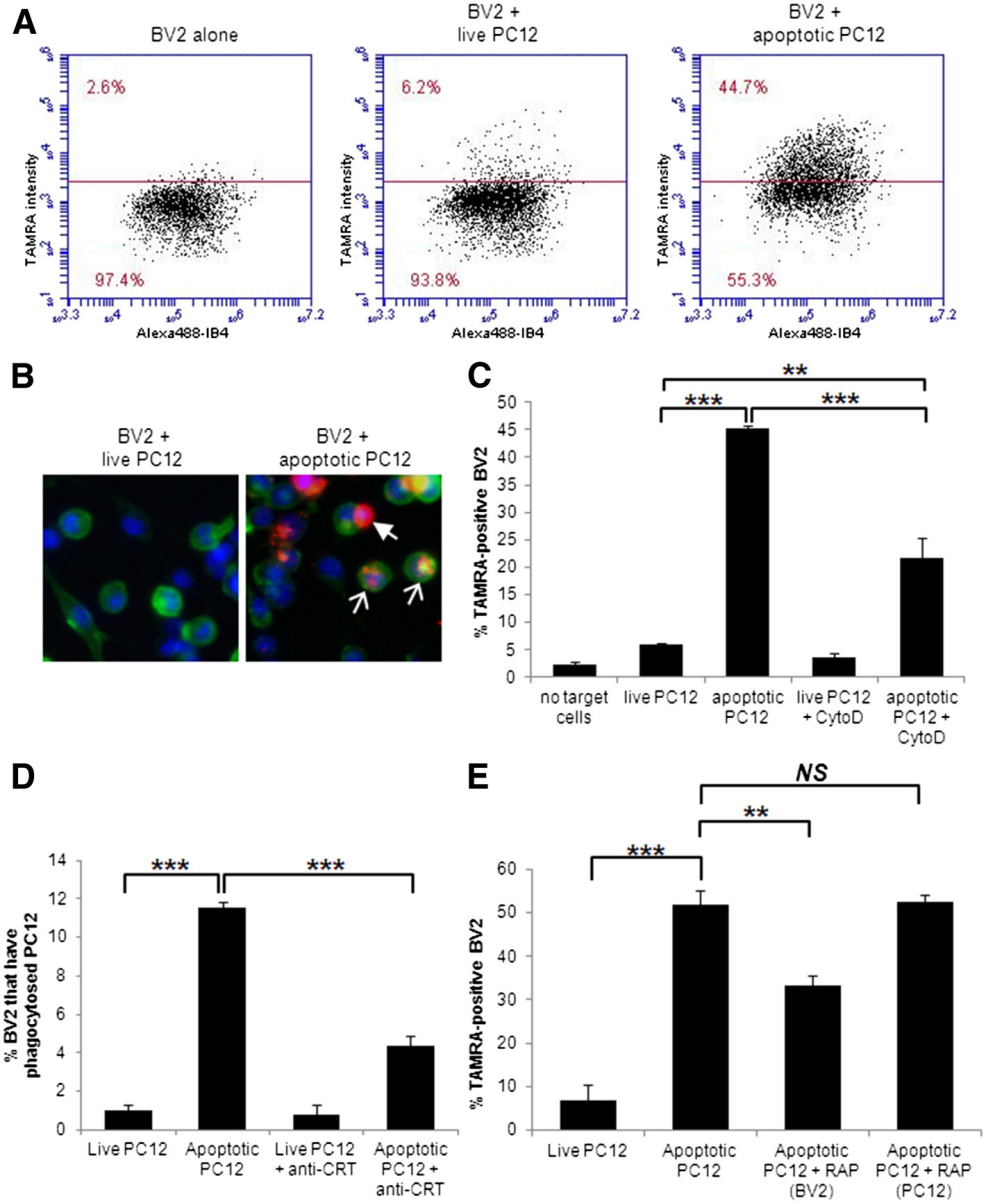
**Phagocytosis of apoptotic neurons by microglia requires LRP/CRT signalling. (A)** Example of flow cytometer phagocytosis assay data. Points represent individual cells with on the X axis staining for Alexa 488 IB4 (bound to BV2 cells only) and on the Y axis staining for TAMRA (bound to PC12 cells only). Co-staining (above the line) indicates BV2 phagocytosis of PC12s, which is increased when PC12s are apoptotic (right). **(B)** Fluorescent microscopy images of BV2 cells labelled with IB4 (green) showing BV2 that have phagocytosed TAMRA-labelled target cells (white hollow arrows) and unphagocytosed TAMRA-labelled target cells attached to BV2 cell surface (white solid arrow). DNA stained with Hoechst 33342 is shown in blue. **(C)** Cytochalasin D inhibits phagocytosis of apoptotic TAMRA-labelled PC12 by BV2 (flow data). **(D)** 10 μg/ml CRT-blocking antibody inhibits phagocytosis of apoptotic PC12 by BV2 (microscopy data). **(E)** Addition of 250 nM RAP to BV2 inhibits phagocytosis, but not when added to the apoptotic target PC12 cells (flow data). (**C**), (**D**) and (**E**) represent mean value from three separate experiments with two replicates per experiment. Error bars represent SEM. CRT, calreticulin; IB4, isolectin B4; LRP, low-density lipoprotein receptor-related protein; RAP, receptor-associated protein; TAMRA, 5-(and-6)-carboxytetramethylrhodamine succinimidyl ester.

Cell-surface-exposed CRT has been demonstrated to play an important role in mediating phagocytosis in various cancer cell lines, either as an eat-me signal on the target cell or as a co-receptor on the phagocytic cell surface. Whether exposed CRT plays a role in phagocytosis of neurons by microglia is unknown. Given the potential importance of phagocytosis of neurons by microglia in modulating neurodegenerative processes, we sought to test whether phagocytosis of neurons by microglia involved CRT-mediated signalling. In order to test the role of CRT in phagocytosis of apoptotic neurons by microglia, we established a phagocytosis assay using the transformed microglial cell line, BV2, and the cell line PC12 as target cells. BV2 cells were visualised with Alexa 488-labelled isolectin B4 (IB4) whilst PC12 cells were separately labelled with red fluorescent 5-(and-6)-carboxytetramethylrhodamine succinimidyl ester (5(6)-TAMRA) prior to treatment and addition to BV2. The extent of phagocytosis was evaluated by determining the proportion of BV2 cells that contained red fluorescence, indicative of phagocytosis of the TAMRA-labelled target cells. Initially, red fluorescence of BV2 was evaluated by fluorescence microscopy and in later experiments FACS analysis was employed (see figure legend for details) (Figure 1A + B). Some experiments were performed using both fluorescence microscopy and flow cytometry for data collection, and flow cytometry was more sensitive to low levels of TAMRA staining within the BV2 cells, so that a higher level of labelled BV2 cells was seen by flow cytometry. However, the trends seen when phagocytic inhibitors were added were similar and statistically significant with both methods (data not shown). As expected very little phagocytosis occurred when live PC12 were added to BV2 (5.9% ± 0.5%, Figure 1C), but this greatly increased when apoptotic PC12 were added (45.3% ± 0.5%, Figure 1C). Cytochalasin D, a well-characterised inhibitor of phagocytosis, significantly inhibited the phagocytosis of apoptotic cells by BV2, demonstrating that phagocytosis of target cells was required for TAMRA uptake by BV2 (Figure 1C). We tested the requirement for cell surface CRT and LRP for phagocytosis of apoptotic cells by microglia using a CRT-blocking antibody [19] and recombinant RAP, a LRP-binding protein, which inhibits ligand binding by LRP [23]. Addition of a CRT-blocking antibody to PC12 cells prior to washing and then addition to BV2 was sufficient to significantly reduce phagocytosis of apoptotic PC12 cells by BV2 (live PC12, 1% ± 0.3%; apoptotic PC12, 11.5% ± 0.3%; apoptotic PC12 + CRT blocking, 4.3% ± 0.5%; Figure 1D). RAP significantly inhibited phagocytosis of apoptotic PC12 when incubated with BV2 prior to addition of target cells but not when incubated with apoptotic PC12 cells prior to addition to BV2 (live PC12, 6.7% ± 3.7%; apoptotic PC12, 52% ± 3.1%; apoptotic PC12 + RAP microglia, 33.2% ± 2.4%; apoptotic PC12 + RAP neuron, 52.7% ± 1.4%; Figure 1E). Thus phagocytosis of apoptotic PC12 by the microglial cell line BV2 requires recognition of CRT on the target cell membrane and ligand binding by LRP on the microglia.

### CRT/LRP signalling in primary phagocytosis of viable cerebellar granule neurons by microglia activated by LPS or Aβ

Tumour cell lines and transformed cells frequently expose the eat-me signal CRT at the cell surface and consequently CRT plays a pivotal role in determining whether or not these cells are phagocytosed [13]. Less is known about the role of CRT in phagocytosis of primary cells, including neurons. We recently demonstrated that in addition to clearance of apoptotic and necrotic debris, in certain circumstances inflammatory-activated microglia may actively participate in neurodegenerative processes by phagocytosing viable neurons both *in vitro* and *in vivo*[5,7]. Inflammatory-activated microglia produce ROS/RNS that induce reversible exposure of PS on the neuronal surface, at which point microglia phagocytose the viable PS-exposing neurons resulting in neuronal death by ‘primary phagocytosis’ [5]. A defining feature of primary phagocytosis as a form of cell death is that inhibition of any of the critical components of the phagocytic signalling machinery is sufficient to rescue neurons, both *in vitro* and *in vivo*.

**Figure 2:**
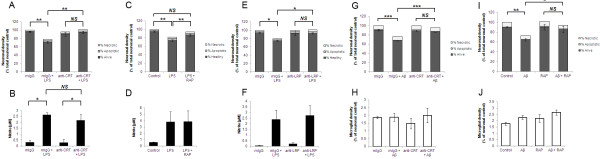
**Disruption of CRT/LRP phagocytic signalling inhibits primary phagocytosis induced by LPS or Aβ. (A, C + E)** Co-cultures of cerebellar neurons and glia were treated with 100 ng/ml LPS for 72 hours in the presence of 1 μg/ml CRT-blocking antibody (**A**), 250 nM RAP (**C**) or 1 μg/ml LRP-blocking antibody (**E**). In (**A**) and (**E**) normal mouse IgG (mIgG) was added to control for non-specific effects of CRT- and LRP-blocking antibodies. In (**C**) and **(I)** ‘control’ refers to addition of PBS alone as RAP was dissolved in PBS prior to addition. Neuronal survival was quantified using Hoechst/PI staining after 72 hours. **(B, D + F)** Production of nitrite as a measure of microglial activation was measured in cell culture supernatants from experiments shown in (**A**) (**B**), (**C**) (**D**) and (**E**) (**F**). (**G**-**J**) Cerebellar co-cultures were treated with 250 nM Aβ peptide for 72 hours in the presence of CRT-blocking antibody (**G + H**) or RAP (**I + J**) prior to quantification of neuronal survival (**G + I**) and microglial density (**H** + **J**). All data represent the mean value of three separate experiments (two replicates per experiment). Error bars represent SEM. Aβ, amyloid-β peptide_1-42_; CRT, calreticulin; LPS, lipopolysaccharide; LRP, low-density lipoprotein receptor-related protein; RAP, receptor-associated protein.

We tested the role of CRT and LRP in primary phagocytosis using the TLR4 ligand LPS as an inflammatory stimulus. LPS has been well characterised by us and others as a means of inducing microglial activation in models of inflammatory neurodegeneration [24]. As previously reported [5], 72 hours of LPS treatment resulted in the disappearance of a portion of healthy neurons without any concurrent appearance of dead neurons (Figure 2A). LPS-induced neuronal death was entirely dependent on the presence of microglia in the culture (data not shown and [5]). Addition of a CRT-blocking antibody was sufficient to prevent neuronal death, without blocking microglial activation (as indicated by increased nitrite production in the presence of LPS and microglial proliferation) (Figure 2A + B and data not shown). Similarly, the LRP-blocking protein RAP significantly inhibited LPS-induced neuronal death without affecting microglial nitrite production (Figure 2C + D). We also tested a LRP-blocking antibody [19] that provided almost complete rescue from LPS-induced neuronal death and had no effect on microglial activation as measured by nitrite production (Figure 2E + F). Isotype-matched IgG controls had no effect on LPS-induced neuronal loss and microglial activation (2A, B, E + F).

We recently reported that nanomolar concentrations of amyloid-beta (Aβ) peptide that were not directly neurotoxic were able to induce primary phagocytosis of neurons by microglia in a PS-dependent manner [6]. Of note, Aβ-induced primary phagocytosis is not accompanied by the usual markers of microglial activation such as nitrite production, TNF-α release and microglial proliferation, potentially reflective of a M2-type microglial phenotype [25]. We tested whether CRT/LRP phagocytic signalling may also be required for Aβ-induced primary phagocytosis using the CRT-blocking antibody and RAP. As expected, Aβ induced a significant loss of neurons after 72 hours treatment, without increasing the number of apoptotic or necrotic neurons (Figure 2G-J). However, addition of either CRT-blocking antibodies or LRP-blocking RAP was able to prevent Aβ-induced primary phagocytosis and rescue viable neurons (Figure 2G-J). In sum, inhibition of the CRT/LRP phagocytic machinery was sufficient to prevent primary phagocytosis of neurons by microglia induced by either LPS or Aβ.

### CRT acts as an ‘eat-me-if’ signal on neurons

**Figure 3:**
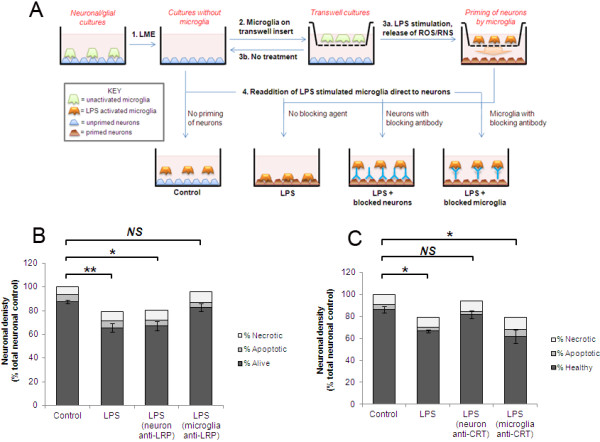
**LRP blocking agents act on microglia and CRT blocking inhibits neuronal sites to prevent primary phagocytosis. (A)** Schematic showing experimental design for microglial elimination/reconstitution/inhibition studies. **(B)** 1 μg/ml LRP-blocking antibody inhibits LPS-induced primary phagocytosis of PS-exposing ‘primed’ neurons when antibody added to microglia but not when incubated with neuronal targets prior to readdition of microglia (excess antibody was washed off cells prior to addition of microglia to neurons). Microglia were added to primed neuronal cultures for six hours followed by quantification of neuronal survival. **(C)** 1 μg/ml CRT-blocking antibody inhibits LPS-induced primary phagocytosis when pre-incubated with primed neurons but not when pre-incubated with microglia. (**B**) and (**C**), data represent three separate experiments with two replicates per experiment. mIgG = mouse IgG control. Error bars represent SEM. CRT, calreticulin; LPS, lipopolysaccharide; LRP, low-density lipoprotein receptor-related protein; PS, phosphatidylserine.

The above data indicated that CRT/LRP were necessary for primary phagocytosis of cerebellar granule neurons by microglia to occur. As discussed above, two roles for surface-exposed CRT have been described, one as an eat-me signal on the target cell surface, acting as a binding ligand for LRP on the phagocytic cell (described as *trans*) and a second role with CRT acting as a co-receptor with LRP for other ligands including complement component C1q (*cis*) on the phagocyte membrane [15,18]. We sought to test the site of actions of both CRT and LRP blocking agents and thus establish whether CRT/LRP were operating in *trans* or *cis* during primary phagocytosis. We previously demonstrated that microglia placed in a transwell were able to release ROS/RNS upon activation and induce reversible PS flip of viable neurons, although physical contact was required for primary phagocytosis to proceed [5]. These ‘primed’ PS-exposing neurons were then rapidly phagocytosed when activated microglia were added back directly to the neurons. We used this model to test whether blocking agents would prevent primary phagocytosis if added to primed neurons or if added to activated microglia prior to mixing of the two cell types (Figure 3A for schematic). Following addition of blocking agents, both neurons and microglia were washed to remove excess unbound blocking agent before microglia were added back to the primed neurons. The LRP-blocking antibody prevented primary phagocytosis when added to microglia but not when added to primed neurons, consistent with LRP being required on the microglial/phagocyte surface (Figure 3B). Microglial counts showed that similar amounts of activated microglia were added back to primed neuronal cultures in all conditions (data not shown). In contrast to the LRP-blocking antibody, the CRT-blocking antibody displayed a significant neuroprotective effect when added directly to primed neurons and no protective effect when added to microglia (Figure 3C). Thus the data indicate that CRT/LRP are required and operate in *trans* for primary phagocytosis to proceed.

**Figure 4:**
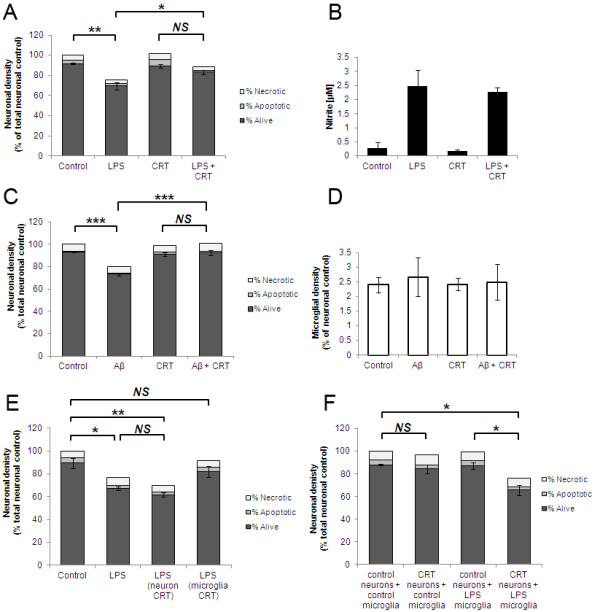
**Exogenous CRT mediates a dominant inhibitory effect on primary phagocytosis by acting on microglia.** (**A + B)** Co-cultures of cerebellar neurons and glia were incubated with LPS for 72 hours in the presence or absence of 2 μg/ml exogenous CRT prior to quantification of neuronal survival (**A**) and microglial activation as measured by nitrite levels in culture supernatant at 72 hour timepoint (**B**). **(C + D)** Co-cultures were incubated with Aβ peptide for 72 hours in the presence or absence of exogenous CRT prior to quantification of neuronal survival (**C**) and microglial density (**D**). **(E) **Exogenous CRT inhibits LPS-induced primary phagocytosis when pre-incubated with microglia but not with primed neurons. CRT was added to either neurons or microglia separately, washed out and then microglia were added to neurons for six hours prior to quantification of neuronal survival (**E**). (**F**) Co-cultures were treated with LME to remove microglia prior to addition of exogenous CRT where indicated. Pure microglia, either unactivated or LPS-activated, were then added back to neurons for six hours prior to quantification of neuronal survival. (**A**-**E**) Data represent three experiments with two replicates per experiment. (**F**) Data represent six experiments with two replicates per experiment. All error bars represent SEM. Aβ, amyloid-β peptide_1-42_; CRT, calreticulin; LME, L-leucine methyl ester; LPS, lipopolysaccharide.

Our data indicated that CRT exposure on the neuronal cell surface was required for primary phagocytosis to proceed. We next tested whether exposed CRT alone was sufficient to allow primary phagocytosis to occur. Several other groups have shown that addition of exogenous CRT to cells is sufficient to allow or enhance phagocytosis of target cells. Intriguingly addition of exogenous CRT to LPS-treated CGC cultures resulted in a significant inhibition of primary phagocytosis, rather than an enhancement as hypothesised (Figure 4A). Inflammatory activation of microglia as measured by nitrite production was not affected by exogenous CRT (Figure 4B). Addition of exogenous CRT also inhibited Aβ-induced primary phagocytosis (Figure 4C + D). We tested whether the inhibitory effect of exogenous CRT was mediated by an action on the neuronal target cells or on the microglia using the method described in Figure 3A. When exogenous CRT was added to primed neurons prior to readdition of activated microglia there was a slight increase in primary phagocytosis, although this did not reach statistical significance (Figure 4E). However, incubation of exogenous CRT with the activated microglia prior to adding them back to the primed neurons significantly inhibited primary phagocytosis (Figure 4E). Thus, exogenous CRT exerted a dominant inhibitory effect on primary phagocytosis when added to microglia.

To test whether surface CRT was sufficient to induce phagocytosis of unprimed neurons we eliminated microglia from CGC with LME, incubated neurons with exogenous CRT, washed off any excess unbound CRT and then added back microglia that were either unactivated or LPS-activated. Addition of exogenous CRT to neurons followed by unactivated microglia did not result in any significant change in numbers of healthy or dead neurons. In contrast, when LPS-activated microglia were added back to neurons that had been incubated with exogenous CRT a significant loss of neurons was observed (unprimed neurons + LPS microglia, 87.4% ± 2.9%; unprimed neurons + exogenous CRT + LPS microglia, 65.9% ± 4.5%; Figure 4F). Thus addition of exogenous CRT to unprimed neurons alone was not sufficient to allow primary phagocytosis, except when microglia had been inflammatory-activated with LPS.

**Figure 5:**
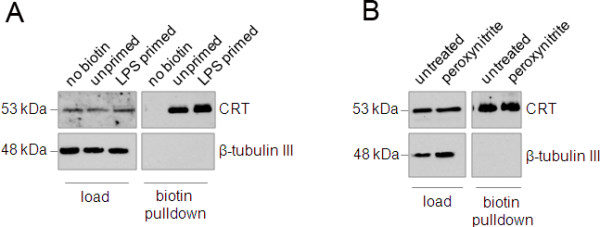
**Constitutive exposure of CRT at the neuronal surface is not modulated during priming of neurons. (A)** Surface biotinylation of primed neurons (see Figure 1A) reveals that CRT is externalised to equal extent on both primed and unprimed neurons. **(B)** Peroxynitrite, which induces PS flip on viable neurons, does not alter surface expression of CRT on neurons. CRT, calreticulin; PS, phosphatidylserine.

We previously demonstrated that inflammatory-activated microglia release ROS/RNS which induce a reversible exposure of PS on the surface of viable neurons [5]. To test whether CRT was exposed in a similar manner to PS, we performed a surface biotinylation of ‘unprimed’ or ‘primed’ neurons (see Figure 3A) and compared levels of biotinylated (that is, externalised) CRT between the two populations. As expected, beta-tubulin was not biotinylated indicating that intracellular proteins were not biotinylated (Figure 5A). In contrast, CRT was biotinylated in both unprimed and primed neuronal cultures, and the amount of biotinylated CRT pulled down did not differ between unprimed and primed neurons. We previously found that treatment with sub-lethal doses of peroxynitrite could induce reversible PS flip on viable neurons but this treatment also had no effect on surface CRT levels on the neurons (Figure 5B). In sum, neurons appeared to expose a constant amount of CRT even in the presence of stimuli that had previously been shown to induce PS flip and allow primary phagocytosis to proceed.

## Discussion

In this study we demonstrate that the CRT/LRP system is required for primary phagocytosis of viable neurons by microglia, so that inhibition of this system could prevent neuronal loss and death induced by LPS or Aβ. Given the increasing evidence supporting a neurodegenerative role for microglia, this system might potentially play a role in loss of neurons during inflammatory neurodegenerative processes such as brain infections (for example, AIDS dementia), ischaemia (for example, stroke), inflammation (for example, multiple sclerosis), trauma or neurodegeneration (for example, Alzheimer’s or Parkinson’s disease) [3,26]. However, this requires further investigation in relevant models of disease. We found that RAP can prevent neuronal loss induced by LPS or Aβ, and thus in principal might be therapeutically useful, at least in acute, life-threatening brain pathologies such as stroke, trauma or meningitis. RAP is normally expressed in the brain but declines in Alzheimer’s disease [27], which might in principle contribute to the neuronal loss.

We have previously shown that microglia activated by LPS or Aβ induce neuronal loss and death by phagocytosis of otherwise viable neurons, and this ‘primary phagocytosis’ required microglia-induced PS exposure by neurons, bound by MFG-E8, which induced phagocytosis of the neurons via microglial vitronectin receptors [5-7]. PS exposure on viable neurons was induced by peroxynitrite production by microglia and phagocytosis of neurons occurred independently of apoptosis [5,7]. In the present work, we have found that the CRT/LRP pathway plays a permissive role for this induced primary phagocytosis, such that if the CRT/LRP pathway is blocked primary phagocytosis can not proceed. The finding that blocking this phagocytic pathway with CRT antibodies, LRP antibodies, RAP or free CRT results in the accumulation of live rather than dead neurons, again supports the notion that the neurons are lost by primary phagocytosis, rather than phagocytosis secondary to the neurons dying by some other means.

We also found that CRT/LRP was important for BV2 microglial phagocytosis of apoptotic PC12 cells. If this is true for primary neurons and microglia *in vivo*, then blocking this system may be detrimental in a variety of physiological and pathological conditions by allowing dead neurons to accumulate and promote inflammation. However, for cancer cells, it has been shown that macrophage phagocytosis of CRT-exposed cancer cells is immunogenic as the macrophages present antigens from these cells [14]. This immunogenic phagocytosis of CRT exposing dead cells might be beneficial in the context of brain tumours or brain infections, but potentially detrimental in other contexts such as MS or development. It is possible that the CRT/LRP system contributes to the phagocytosis apoptotic and viable neurons arising during development. In *C.elegans* mutation of a number of the *Ced* genes (including *Ced1,* a proposed functional homologue of LRP) involved in phagocytosis of dead cells in combination with a weak *Ced3* (caspase) mutation results in survival of cells normally eliminated in the presence of the weak *Ced3* mutation alone [28,29]. CRT knockout is lethal in mice, and intriguingly 16% of CRT-null mice develop exencephaly of the brain characterised by failure to close the neural tube, a process involving programmed cell death [30,31].

Our data are consistent with a model in which CRT acts as an eat-me signal on the neuronal surface and is recognised by LRP on the phagocytic membrane, as has been described in other non-neuronal systems [15,17]. LRP is known to be expressed and functional on microglia [32]. Through use of surface biotinylation, we demonstrated that CRT is constitutively expressed on the surface of cerebellar neurons. A previous report from Hossain and colleagues demonstrated external localisation of CRT on rat hippocampal neurons where it co-localised with NMDA receptor and potentially played a role in modulating Ca^2+^ influx into neurons [33]. The amount of CRT was unchanged when neurons were primed for phagocytosis by inflammatory-activated microglia. Studies in non-neuronal cell types have shown that CRT-dependent phagocytosis does not necessarily require increased CRT exposure. In some cell types surface-exposed CRT accumulates in patches on the cell surface, often in association with exposed PS [15,16,34]. CRT-dependent phagocytosis can also be triggered by a reduction in don’t-eat-me signalling by CD47/SIRPα signalling [13,15,19]. However, we tested a CD47-blocking antibody and found that this had no effect on phagocytosis of viable or apoptotic neuronal cells in the presence or absence of inflammatory stimuli (data not shown). Gardai and colleagues demonstrated that CRT knockout prevented phagocytosis of PS-exposing apoptotic cells, and that readdition of exogenous CRT restored phagocytosis although this phagocytosis remained PS-dependent [15]. Similarly, in our model we have shown that LPS-activated microglia induce PS exposure on viable neurons and that PS recognition by MFG-E8 is required for phagocytosis to proceed [5,7]. Thus whilst CRT is not increased on the surface of viable neurons during primary phagocytosis, the exposure of PS induced by LPS-activated microglia may be sufficient to allow phagocytosis that occurs in a CRT-dependent manner. We demonstrated that incubation of viable neurons with exogenous CRT allowed primary phagocytosis to proceed in the presence of activated but not unactivated microglia. It is possible that other types of neuropathologically relevant stimuli may cause increased neuronal CRT exposure and, therefore, may cause neurodegeneration by primary phagocytosis in this way.

We found that addition of CRT to microglia or to both microglia and neurons could block microglia-induced neuronal loss. This may be because free CRT can activate microglial phagocytosis/endocytosis via LRP in the absence of bound neurons/target cells (as occurs in macrophages [15]), resulting in downregulation of surface LRP and associated phagocytic machinery. This type of mechanism might be involved in the neuroprotective effect of peptide Y-P30. Y-P30 can bind CRT and was reported to cause release of extracellular CRT in SH-SY5Y cells, as well as dissociation of CRT from membranes isolated from rat cortex. In conjunction with this activity, Y-P30 inhibited the appearance of microglia *in vivo* following lesioning of the cortex in rat [35].

## Conclusions

**Figure 6:**
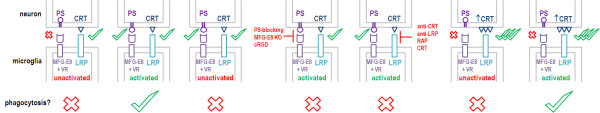
**Schematic representing requirements for primary phagocytosis of neurons to proceed.** Primary phagocytosis of viable neurons does not proceed without microglial activation. Basal levels of surface-exposed CRT are not sufficient alone to allow phagocytosis, but CRT-dependent phagocytosis can be triggered by an increase in either PS or CRT exposure. CRT, calreticulin; PS, phosphatidylserine.

CRT exposure on the surface of viable or apoptotic neurons is required for their phagocytosis via LRP receptors on microglia. CRT is exposed on neurons in the presence or absence of inflammation, and appears to be permissive for PS-induced phagocytosis. Based on our present and previous data, we propose that CRT acts as an ‘eat-me-if’ signal as illustrated in Figure 6. Phagocytosis of neurons by microglia normally requires: i) CRT exposure on neurons, AND ii) PS exposure on neurons, AND iii) activation of microglia. However, high levels of CRT exposure on neurons can override the requirement for PS exposure, and free CRT can block phagocytosis of neurons by acting directly on microglia.

## Competing interests

The authors declare they have no competing interests.

## Authors’ contributions

MF performed surface biotinylation, primary tissue culture and treatments, helped design the study and drafted the manuscript. MJOM performed apoptotic phagocytosis assays. GCB conceived and directed the study and helped draft the manuscript. All authors read and approved the final manuscript.
